# Tumor-associated neutrophils correlate with poor prognosis in diffuse large B-cell lymphoma patients

**DOI:** 10.1038/s41408-018-0099-y

**Published:** 2018-07-05

**Authors:** B. Manfroi, J. Moreaux, C. Righini, F. Ghiringhelli, N. Sturm, B. Huard

**Affiliations:** 1 Institute for Advanced Biosciences, INSERM U 1209, University Grenoble-Alpes, La Tronche, France; 2 Department of Biological Hematology, Institute of Human Genetics, CNRS-UM UMR 9002, CHU Montpellier, University of Montpellier, UFR de Médecine, Montpellier, France; 3 Head and Neck Department, University Hospital of Grenoble, La Tronche, France; 4 Centre Georges François Leclerc, INSERM U1231, University Bourgogne Franche Comté, Dijon, France; 5 Department of Anatomopathology and Cytology, University Hospital of Grenoble, La Tronche, France

Neutrophil is a myeloid cell that has been overlooked in tumor biology. Recent studies indicated that neutrophil infiltration may modulate tumor prognosis either by exerting a pro- or an anti-tumoral effect^1^. Tumor-associated neutrophils (TANs) may be involved in tumor promotion and development by increasing genomic instability through reactive oxygen species (ROS), inducing immunosuppression through secretion of arginase-1/interleukin (IL)-10, and angiogenesis increase. For all these reasons, neutrophil infiltration has often been associated with adverse events in various solid cancers^2^. On the other side, TAN can be involved in tumor regression by inducing tumor cell death through ROS production, by their expression of the apoptotic ligand from the tumor necrosis factor superfamily, TRAIL, and their capacity to mediate antibody-dependent cell cytotoxicity^1^.

We recently reported that a significant fraction of diffuse large B-cell lymphoma (DLBCL), an aggressive neoplasm derived from germinal center experienced B cells, specifically recruits in a CXCL-8/IL-8-dependent manner blood neutrophils^3^. In DLBCL patients, all TAN produced the pro-tumoral factor, a proliferation-inducing ligand and expression of this tumor cell survival factor allowed the identification of patients with poor prognosis in the CHOP (Cyclophosphamide, Hydroxydaunorubicin, Oncovin, and Prednisone) era^4^. However, the overall function of TAN on DLBCL development remains elusive.

To establish the impact of TAN on DLBCL patient survival, we took advantages of available gene expression data and associated clinical data. Based on our previous in situ studies, we used the neutrophil elastase (*ELANE*) gene as a marker of TAN infiltration. A first discovery cohort of 233 DLBCL patients treated with the gold standard chemo-immunotherapy R-CHOP (Rituximab+CHOP) was analyzed with GenomicScape online data-mining platform (accession number GSE10846)^5,6^. MaxStat function in R software was used to determine the optimal cut point for continuous variables^7^. The discovery cohort highlighted a high level of *ELANE* expression in 81.5% of patients and was associated with a reduced overall survival (hazard ratio (HR) 2.3, 95% confidence interval (CI) 1.2–4.3, *p* = 0.01) (Fig. 1a). These results were reproduced in three independent validation cohorts of R-CHOP-treated DLBCL patients, available under accession numbers GSE32918, GSE53786, and GSE23501^8–10^. Patients with a high *ELANE* expression also demonstrated a poorer overall survival (Fig. 1b). This was true for cohort GSE32918 (HR 2.6, 95% CI 1.0–7.4, *p* = 0.007), GSE53786 (HR 2.0, 95% CI 1.2–3.3, *p* = 0.05), and GSE23501 (HR 4.5, 95% CI 1.4–13.8, *p* = 0.01). Thresholds determined with MaxStat function allowed us to identify patients with a high level of *ELANE* expression in 50.7%, 58.6%, and 73.9% of cohorts GSE32918, GSE53786, and GSE23501, respectively. This high level of *ELANE* expression is in accordance with our previous in situ studies at the protein level reporting between 50% and 75% DLBCL tumor lesions infiltrated by TAN^3,4^. We then investigated whether *ELANE* expression related to TAN infiltration provided additional prognostic information compared with described outcome-related factors such as the germinal center B-cell like (GCB) and activated B-cell like (ABC) molecular subgroups, age, index prognostic international (IPI), and Gene Expression-based Risk Score (GERS)^11^. *ELANE* expression, age, GCB–ABC molecular subgroups, IPI, and GERS had a prognostic value in the Lenz R-CHOP cohort (*n* = 233 patients) (Supplementary Table 1A). When tested all together only ELANE expression, GERS, and GCB–ABC molecular subgroups remained independent prognostic factors (Supplementary Table 1B). Furthermore, *ELANE* expression was not significantly different when comparing ABC and GCB DLBCL patients. In addition, no significant differences in *ELANE* expression were identified between DLBCL patients classified according to IPI (Supplementary Figure 1). Considering the in vitro promoting role exerted by neutrophils on DLBCL tumor cells^12^, our data demonstrated that infiltrating neutrophils have a DLBCL tumor-promoting role, strong enough to modulate patient survival

**Fig. 1:**
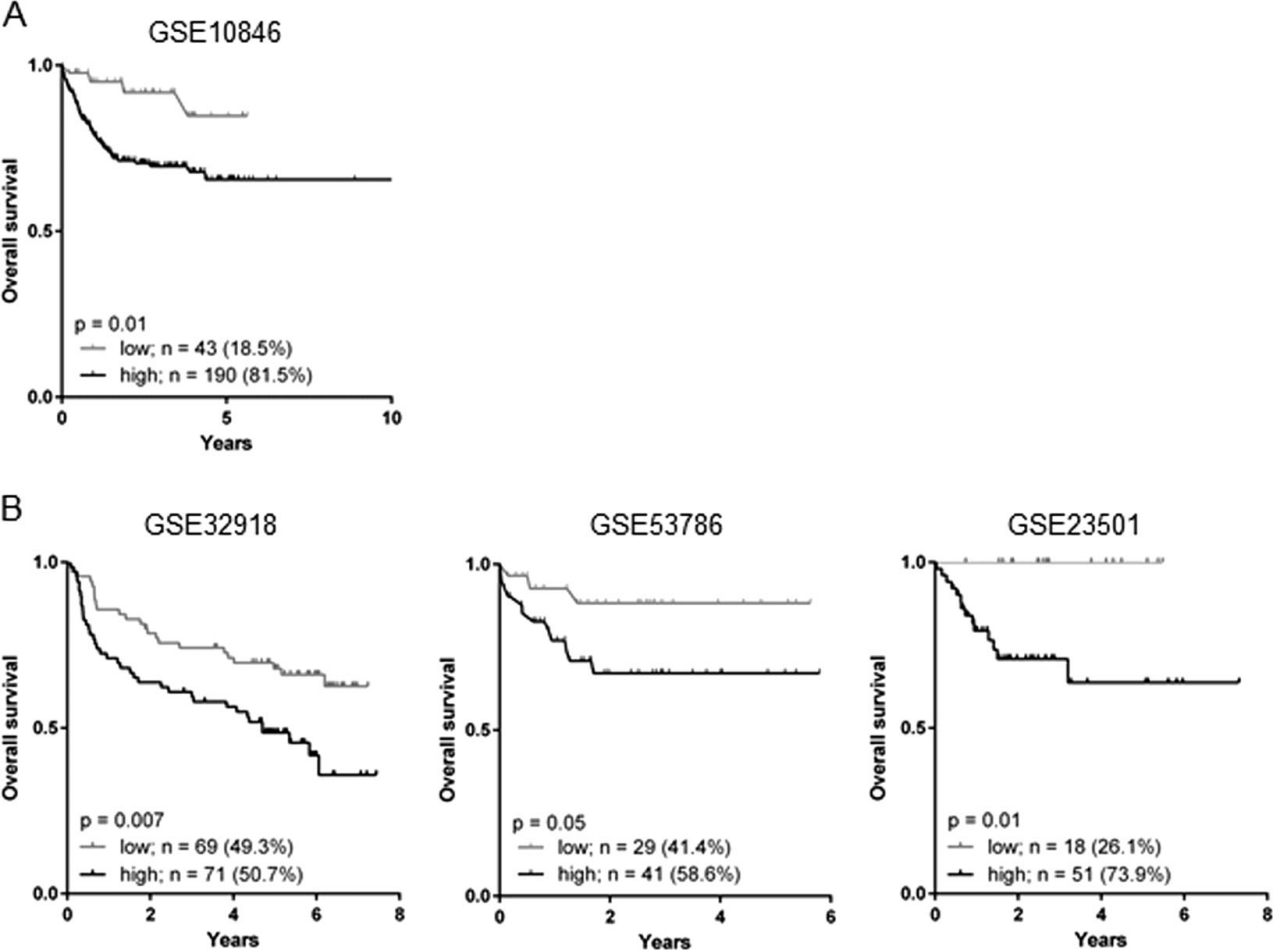
Neutrophil gene signature correlates with poor prognosis in R-CHOP treated DLBCL patients **a** Discovery dataset GSE10846 was analyzed for prognosis value of neutrophil elastase (*ELANE*) expression on R-CHOP-treated patient’s overall survival. **b** Validation datasets from R-CHOP-treated patients GSE32918 (left panel), GSE53786 (middle panel), and GSE23501 (right panel) were analyzed as in **a**. Thresholds were determined with MaxStat function in R software. Data are presented as Kaplan–Meier curves and compared with log-rank test (GraphPad Prism 6)

When one looks at myeloid cells in cancer patients, an immediate interrogation arises regarding their nature. In other words, are these cells myeloid-derived suppressor cells with an immature phenotype and arising from emergency myelopoiesis or represent purely mature cells originating from steady-state hematopoiesis? This dichotomy has been evidenced for mononuclear monocyte/macrophage myeloid-derived suppressor cells (MDSCs) but also polymorphonuclear granulocyte-MDSCs (PMN-MDSCs). According to the maturation pattern of granulocytes, surface expression of major histocompatibility complex (MHC) class II molecules represents immature cells^13^. In addition, expression of the LDL receptor, Lox1, in granulocytes has also been recently associated to granulocyte immaturity^13^. We assessed MHC Class II (Mouse IgG_1_, clone CR3/43, Dako) and Lox1 (Goat polyclonal IgG, ThermoFisher) protein expression in situ by immunofluorescence assays. Because of the lack of compatibility for elastase heat-induced epitope retrieval with above-mentioned markers, we used here CD15 as a neutrophil marker. We never found CD15^+^ TAN costained for MHC class II nor for Lox1 in DLBCL lesions (Fig. 2a). In fact, DLBCL lesions were devoid of Lox1^+^ cells. We found Lox1^+^ PMN-like CD15^+^ cells, but it was in blood vessels from healthy secondary lymphoid organs (Fig. 2b). In various cancers, PMN-MDSC have impaired migratory properties due to decreased expression of CXCR-1 and CXCR-2, the two CXCL-8-receptors^14^. As TAN are selectively attracted in DLBCL lesions by a CXCL-8-mediated mechanism^3^, our work strongly argue for a mature phenotype of TAN in DLBCL. Blood data already indicated that increased neutrophil-to-lymphocyte ratio was associated to poor DLBCL patient prognosis^15^. The overall DLBCL-promoting activity reported here for neutrophils indicate that TAN may represent an attractive therapeutic target by interfering with the CXCL-8/CXCR-1, -2 chemotactic pathways, already demonstrated to be druggable.

**Fig. 2:**
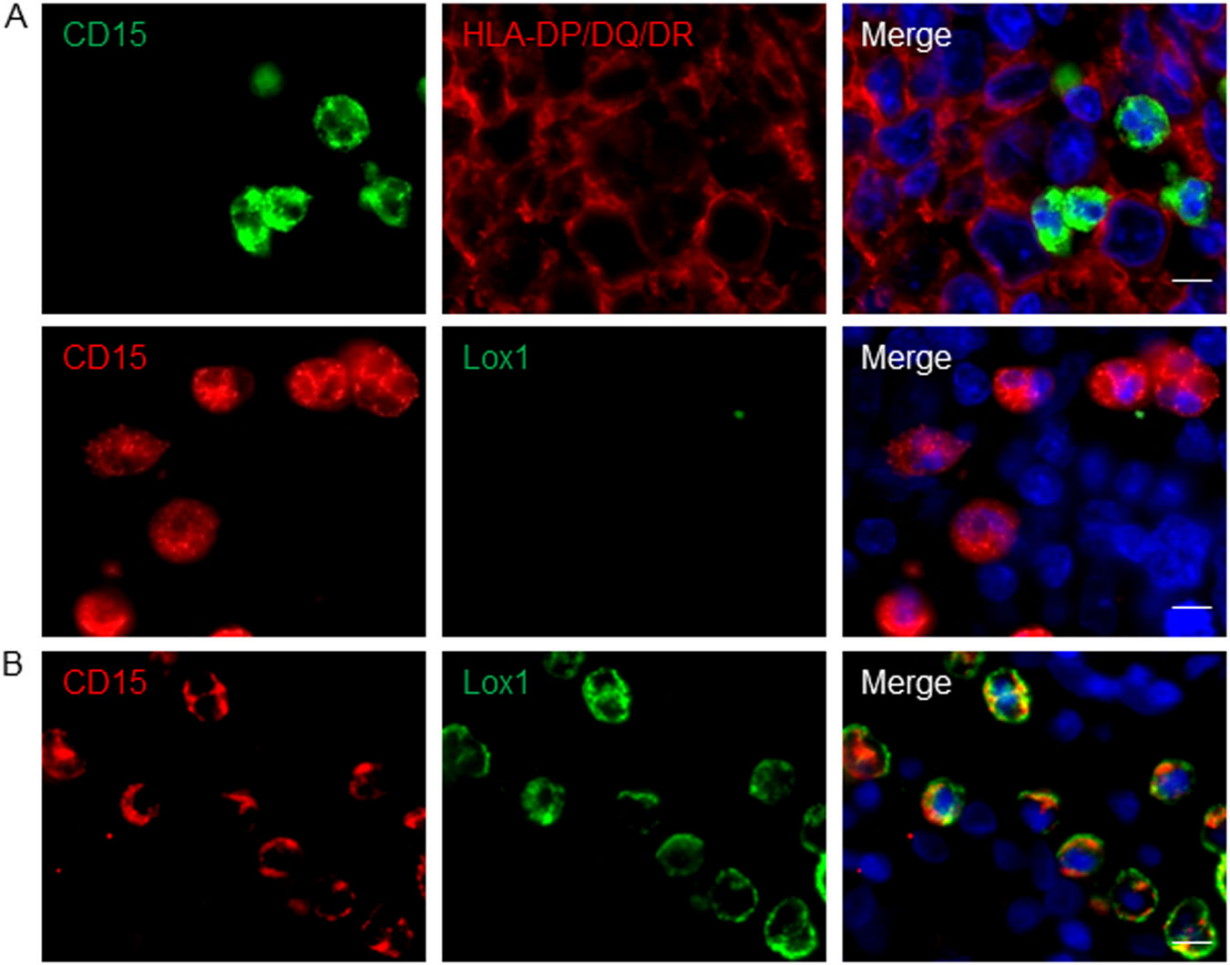
Tumor-associated neutrophils from DLBCL patients display a mature blood neutrophil phenotype **a** In situ fluorescence co-staining of DLBCL biopsies for CD15 and HLA-DR (upper panel) or Lox1 (lower panel). **b** In situ fluorescence co-staining of tonsil for Lox1 and CD15. Hoechst33342 nuclear staining (blue) is also shown in the merge pictures. Pictures are representative of 10 DLBCL patients and three healthy tonsils. Scale bar = 10 µm

## Electronic supplementary material

Supplementary figure 1

Supplementary table 1

## References

[1] Uribe-Querol E, Rosales C (2015). Neutrophils in cancer: two sides of the same coin. J. Immunol. Res..

[2] Shen M (2014). Tumor-associated neutrophils as a new prognostic factor in cancer: a systematic review and meta-analysis. PLoS ONE.

[3] Manfroi B (2017). CXCL-8/IL8 produced by diffuse large B-cell lymphomas recruits neutrophils expressing a proliferation-inducing ligand APRIL. Cancer Res..

[4] Schwaller J (2007). Neutrophil-derived APRIL concentrated in tumor lesions by proteoglycans correlates with human B-cell lymphoma aggressiveness. Blood.

[5] Kassambara A (2015). GenomicScape: an easy-to-use web tool for gene expression data analysis. Application to investigate the molecular events in the differentiation of B cells into plasma cells. PLoS Comput. Biol..

[6] Lenz G (2008). Stromal gene signatures in large-B-cell lymphomas. N. Engl. J. Med..

[7] Hothorn T, Lausen B (2003). On the exact distribution of maximally selected rank statistics. Comput. Stat. Data Anal..

[8] Shaknovich R (2010). DNA methylation signatures define molecular subtypes of diffuse large B-cell lymphoma. Blood.

[9] Scott DW (2014). Determining cell-of-origin subtypes of diffuse large B-cell lymphoma using gene expression in formalin-fixed paraffin-embedded tissue. Blood.

[10] Barrans SL (2012). Whole genome expression profiling based on paraffin embedded tissue can be used to classify diffuse large B-cell lymphoma and predict clinical outcome. Br. J. Haematol..

[11] Bret C, Klein B, Moreaux J (2012). Gene expression-based risk score in diffuse large B-cell lymphoma. Oncotarget.

[12] Gregoire M (2015). Neutrophils trigger a NF-kappaB dependent polarization of tumor-supportive stromal cells in germinal center B-cell lymphomas. Oncotarget.

[13] Condamine T (2016). Lectin-type oxidized LDL receptor-1 distinguishes population of human polymorphonuclear myeloid-derived suppressor cells in cancer patients. Sci. Immunol..

[14] Brandau S (2011). Myeloid-derived suppressor cells in the peripheral blood of cancer patients contain a subset of immature neutrophils with impaired migratory properties. J. Leukoc. Biol..

[15] Wang J, Zhou X, Liu Y, Li Z, Li X (2017). Prognostic significance of neutrophil-to-lymphocyte ratio in diffuse large B-cell lymphoma: A meta-analysis. PLoS ONE.

