# Supplementary figures and images for: Tumor-associated neutrophils correlate with poor prognosis in diffuse large B-cell lymphoma patients
Source: Blood Cancer J. 2018 Jul 5;8(7):66. doi: 10.1038/s41408-018-0099-y (PMC6033870; doi:10.1038/s41408-018-0099-y)

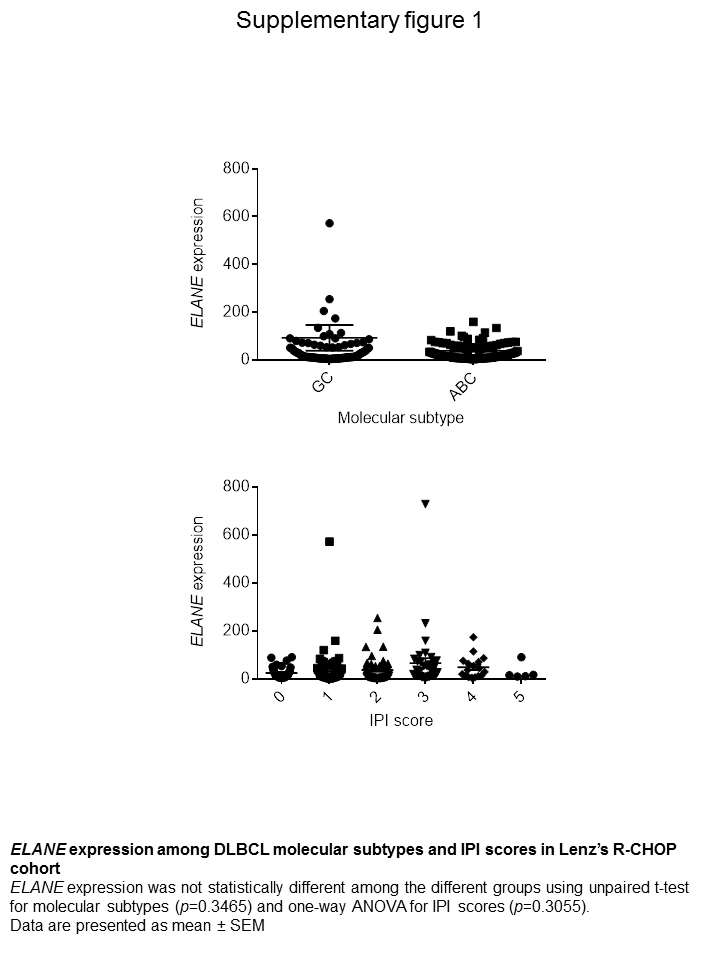

Supplement: Supplementary file 1 — Supplementary figure 1 [file 41408_2018_99_MOESM1_ESM.tif]

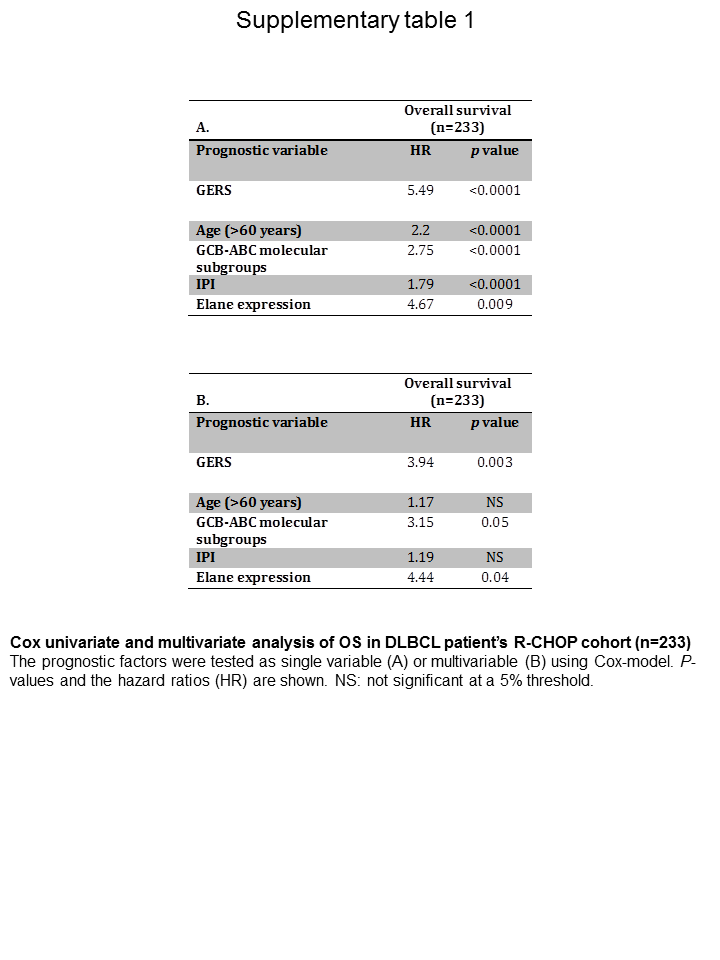

Supplement: Supplementary file 2 — Supplementary table 1 [file 41408_2018_99_MOESM2_ESM.tif]
